# Fine scale Spatial-temporal cluster analysis for the infection risk of *Schistosomiasis japonica* using space-time scan statistics

**DOI:** 10.1186/s13071-014-0578-3

**Published:** 2014-12-10

**Authors:** Feng-hua Gao, Eniola Michael Abe, Shi-zhu Li, Li-juan Zhang, Jia-Chang He, Shi-qing Zhang, Tian-ping Wang, Xiao-nong Zhou, Jing Gao

**Affiliations:** Anhui Provincial Institute of Schistosomiasis Control, Hefei, 230061 China; Department of Zoology, Federal University Lafia, P.M. B 146 Lafia, Nasarawa State Nigeria; National Institute of Parasitic Diseases, Chinese Center for Disease Control and Prevention, Key Laboratory of Parasite and Vector Biology, Ministry of Health, Shanghai, 200025 China

**Keywords:** Fine scale spatial-temporal scan statistics, Schistosomiasis japonica, Infection risk analysis, Anhui province

## Abstract

**Background:**

Marching towards the elimination of schistosomiasis in China, both the incidence and prevalence have witnessed profound decline over the past decades, with the strategy shifting from morbidity control to transmission control. The current challenge is to find out hotspots of transmission risk for precise targeted control in low-prevalence areas. This study assessed the risk at the village level, using the spatial and temporal characteristics of *Schistosomiasis japonica* in Anhui province from 2006 to 2012.

**Method:**

The comprehensive database was generated from annual surveillance data at village level in Anhui province between 2006 and 2012, comprising schistosomiasis prevalence among humans and cattle, occurrence rate of infected environments and incidence rate of acute schistosomiasis. The database parameters were matched with geographic data of the study area and fine scale spatial-temporal cluster analysis based on retrospective space-time scan statistics was used to assess the clustering pattern of schistosomiasis. The analysis was conducted by using SaTScan 9.1.1 and ArcGIS 10.0. A spatial statistical modelling was carried out to determine the spatial dependency of prevalence of human infection by using Geoda 1.4.3.

**Result:**

A pronounced decline was found in the prevalence of human infection, cattle infection, occurrence rate of environment with infected vector snails and incidence rate of acute schistosomiasis from 2006 to 2012 by 48.6%, 71.5%, 91.9% and 96.4%, respectively. Meanwhile, all 4 indicators showed a statistically significant clustering pattern both in time and space, with a total of 16, 6, 8 and 4 corresponding clustering foci found respectively. However, the number of clustering foci declined with time, and none was found after year 2010. All clustering foci were mainly distributed along the Yangtze River and its connecting branches. The result shows that there is a direct spatial relationship between prevalence of human infection and the other indicators.

**Conclusion:**

A decreasing trend in space-time clustering of schistosomiasis endemic status was observed between 2006 and 2012 in Anhui province. Nevertheless, giving the complexity in schistosomiasis control, areas within the upper-stream of Yangtze River in Anhui section and its connecting branches should be targeted for effective implementation of control strategies in the future.

## Background

Schistosomiasis japonica, a widespread zoonotic disease transmitted by the parasite *Schistosoma japonicum* is considered a severe risk to public health [1]. This disease was a major concern in China historically because of its high rate of mortality and morbidity, furthermore, about 10 million cases were reported in the early 1950s [2], but concerted control efforts made by the national control programmes and the World bank loan project in the early 21st century made great success [3,4]. Transmission of *S. japonica* was interrupted in five out of 12 historically endemic provinces [5], with the estimated number of cases dropping to 0.8 million [6]. However, due to changing of socio-economic and environmental factors, *S. japonica* remains a serious public health concern in the lake regions, the middle and lower reaches of the Yangtze River of in Hunan, Hubei, Anhui, Jiangxi and Jiangsu provinces, and the mountainous regions in parts of Sichuan and Yunnan provinces in P.R. China [7,8]. To respond to this challenge, this calls for action and a national medium and long-term strategic work plan for schistosomiasis control was launched in 2004 [9,10].

The intensive integrated control strategy aims at national transmission control with focus on the control of infectious sources [11], in addition with other interventions such as agriculture mechanization, environment improving, raising livestock in captivity, grazing prohibition in marshland, reconstructing water supply infrastructure [12,13], routine snail and human surveillance [14,15], the signs of re-emergence of schistosomiasis has been successfully suppressed [16,17]. The estimated number of cases dropped to 0.15 million in 2013 and only 8 acute schistosomiasis cases were reported nationwide [18].

**Figure 1:**
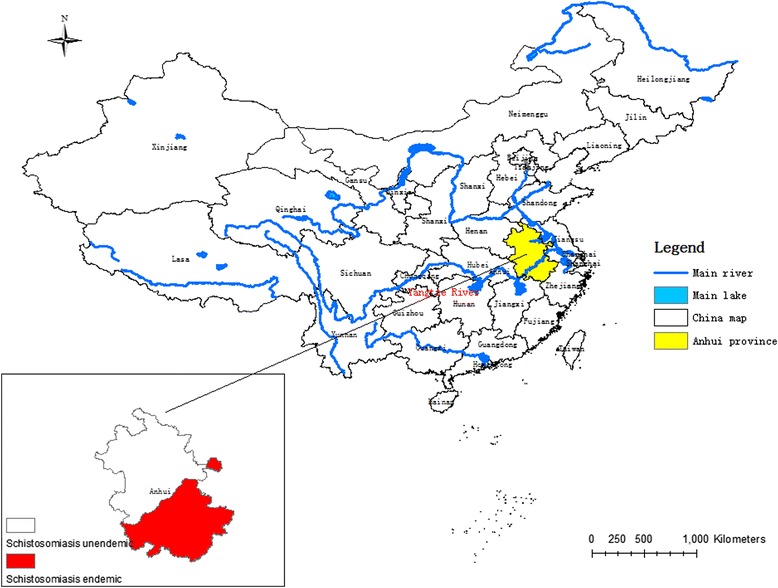
**Map of Schistosomiasis endemic area in Anhui province, China.**

Located in the lower reaches of the Yangtze River, Anhui province has been one of the most severely infected areas for years [19,20] (Figure 1). According to the annual report in 2012, there are still 2401 endemic administrative villages in 51 endemic counties with about 7 million populations at risk in Anhui [21]. A total of estimated 25,378 cases and 5 acute schistosomiasis cases were reported, in which 4 were imported cases. Additionally, among the 1.8 million people screened with IHA for schistosomiasis, 4.4% were positive, while among the 240,000 who were screened using Kato-Katz, eggs of *Schistosoma japonicum* were only found in 0.5% of the population [21]. Cattle were identified as the most important infection source with 23,000 herds screened and 0.4% positive [22]. 6678 spots were detected as suitable areas for snail breeding through surveillance, but only 31(0.5%) were found with infected snails, while 0.29% of the 277 million square meters were sampled harbours infected snails [21]. Therefore, positive declinewas observed in endemic indicators, but the risk factors are still very much in existence. If efficient control measures are not in place, this could trigger re-insurgence of *S. japonica* in the area [22].

In this study, the relationships between human infection rate of schistosomiasis and cattle infection rate, infected vector snails distribution and occurrence of acute schistososmiasis were explored using spatial-temporal scan statistics and a GIS based statistical modelling, to analyze the spatial-temporal clustering of schistosomiasis at village level, thus, to assist our understanding of the current endemic status of schistosomiasis in different areas of Anhui province and provide useful information for further formulation and effective implementation of control strategies.

## Methods

### Study areas

Located in the lower reaches of the Yangtze River, Anhui province used to be severely affected by the epidemic of schistosomiasisin past decadeswith high mortality and morbidity [23]. This study covers all endemic areas in 51 endemic counties in Anhui provinces at village level from 2006 to 2012, including 2401 endemic administrative villages.

### Data collection

From 2005, a governmental subsidized project targeting schistosomiasis control was initiated in Anhui province, which specified the control and surveillance work in each endemic village. Surveillance on humans, livestock and vector snails were performed annually at village level in Anhui province. Residents positive with *S. japonica* infection were further examined using their stool samples (three smears for each sample). Only by finding schistosome eggs in the stool, can etiological diagnosis be made, which is the gold standard for *Schistosomiasis japonicum*. Both the antigen used in IHA analysis and kato-katz kit were produced and provided by the institute of schistosomiasis in Anhui province [24].

A database containing annual surveillance data over a period of 7 years from 2006 to 2012 was set up in a 4-dimensional way: 1) human infection rate, 2) cattle infection rate, 3) acute schistosomiasis and 4) occurrence rate of environment spots with infected vector snails. All data were specific at village level, while acute schistosomiasis was re-distributed to each village based on the location where each case got infected. In the end, 4 indicators were selected and calculated to reflect as follows: Human infection rate (%) = (Number of estimated current schistosomiasis cases/Number of population at risk at village level) * 100%. Estimated current schistosomiasis cases were calculated according to the “Scheme of Investigation and Estimation of Schistosomiasis Cases”, which was published by the Ministry of Health of the People’s Republic of China in 2007 [25]. Cattle infection rate(%) = (Number of positive cattle/Number of cattle being detected) * 100%. Occurrence rate of environment spots with infected vector snails (%) = (Number of environment spots with infected snails/Number of environment spots with snails) * 100%. Incident rate of acute schistosomiasis (1/100,000) = (Number of acute schistosomiasis/Population in endemic village) * 100%.

### Spatial-temporal cluster analysis

The map of schistosomiasis endemic areas in Anhui province was pooled from the County Map of China, with each centimeter in the map representing one meter in real life. However, due to the lack of detailed information on village borders, the location of village committee was selected as the representative in the spatial-temporal analysis, while the specific location was measured by handheld Global Positioning System (GPS). Meanwhile, the pooled map with geographic information was integrated with the aforementioned database containing schistosomiasis endemic status in Anhui province from 2006 to 2012 in ArcGIS 10.0 for further spatial-temporal cluster analysis.

Scan statistics was performed using SatScan software to assess the clustering of prevalence/incidence both in space and time [26], while all 4 indicators were run as an outcome in the analysis separately. The space-time scan statistics was defined by a cylindrical window with a circular geographic base and with height corresponding to time. The window started with the minimum radius and height at one point and in turn centered throughout the confined study areas with continuously varied radius and height until reaching the upper limit. In this study, the upper limit of radius, meaning space, was set at 10% of the total population at risk within the area covered by the window, while the height was set at 50% of the total study period, in years. In the process of centering, Log Likelihood Ratio (LLR) of each potential cluster was formulated based on the calculation of observed and expected prevalence/incidence inside and outside the cylindrical window and a p-value was assigned to it. Those with a p-value being tested less than 0.05 were the clusters indicating accelerated risk of schistosomiasis. Meanwhile, the bigger the LLR, the less likely the cluster detected is due to chance. Besides, Relative Risk (RR) was calculated for each statistically significant cluster, meaning the risk within the cluster in specific time and area compared to the risk outside the cluster.

All the 4 indicators were assumed to follow an independent Poisson distribution. The analytical model chosen to perform the aforementioned analysis was Retrospective Space-Time model based on Poisson distribution. All analysis was performed by using SaTScan v9.1.1, and the Monte Carlo replication was set to 999 times in the model [26,27]. Statistically significant result was considered as *p* value under 0.05. In the end, the results generated from SaTScan9.1.1 were imported to ArcGIS 10.0 and visualized for risk evaluation.

### Spatial regression analysis

The average value of the human infection rate, cattle infection rate, incident rate of acute schistosomiasis and occurrence rate of environment with infected snails of every village were calculated respectively and a new database was formed with every village’s name, longitude and latitude and these four indicators. A software named Geoda 1.4.3 was used to analyze the spatial regression of human infection rate base on the new database [28].

## Results

### Endemic Status of Schistosomiasis in Anhui province from 2006 to 2012

Table 1 shows the human infection rate, cattle infection rate, occurrence rate of environment with infected vector snails and the incidence rate of acute schistosomiasis, it shows a pronounced decrease from 0.7%, 1.93%, 6.2% and 0.84/100,000 in 2006 to 0.36%, 0.55%, 0.5% and 0.03/100,000 in 2012, experiencing a decline of 48.6%、71.5%、91.9% and 96.4%, respectively.

**Table 1:** **Change of endemic status of schistosomiasis in Anhui province, 2006-2012**

**Year**	**Human infection rate (%)**	**Cattle infection rate (%)**	**Occurrence rate of infected environments (%)**	**Incidence rate of acute schistosomiasis (1/100,000)**
2006	0.70	1.93	6.2	0.84
2007	0.61	1.81	4.8	0.26
2008	0.54	1.60	3.9	0.28
2009	0.51	1.25	3.8	0.31
2010	0.48	1.41	3.6	0.27
2011	0.44	0.87	2.6	0.03
2012	0.36	0.55	0.5	0.03

### Spatial temporal cluster analysis of prevalence of schistosomiasis in human

**Figure 2:**
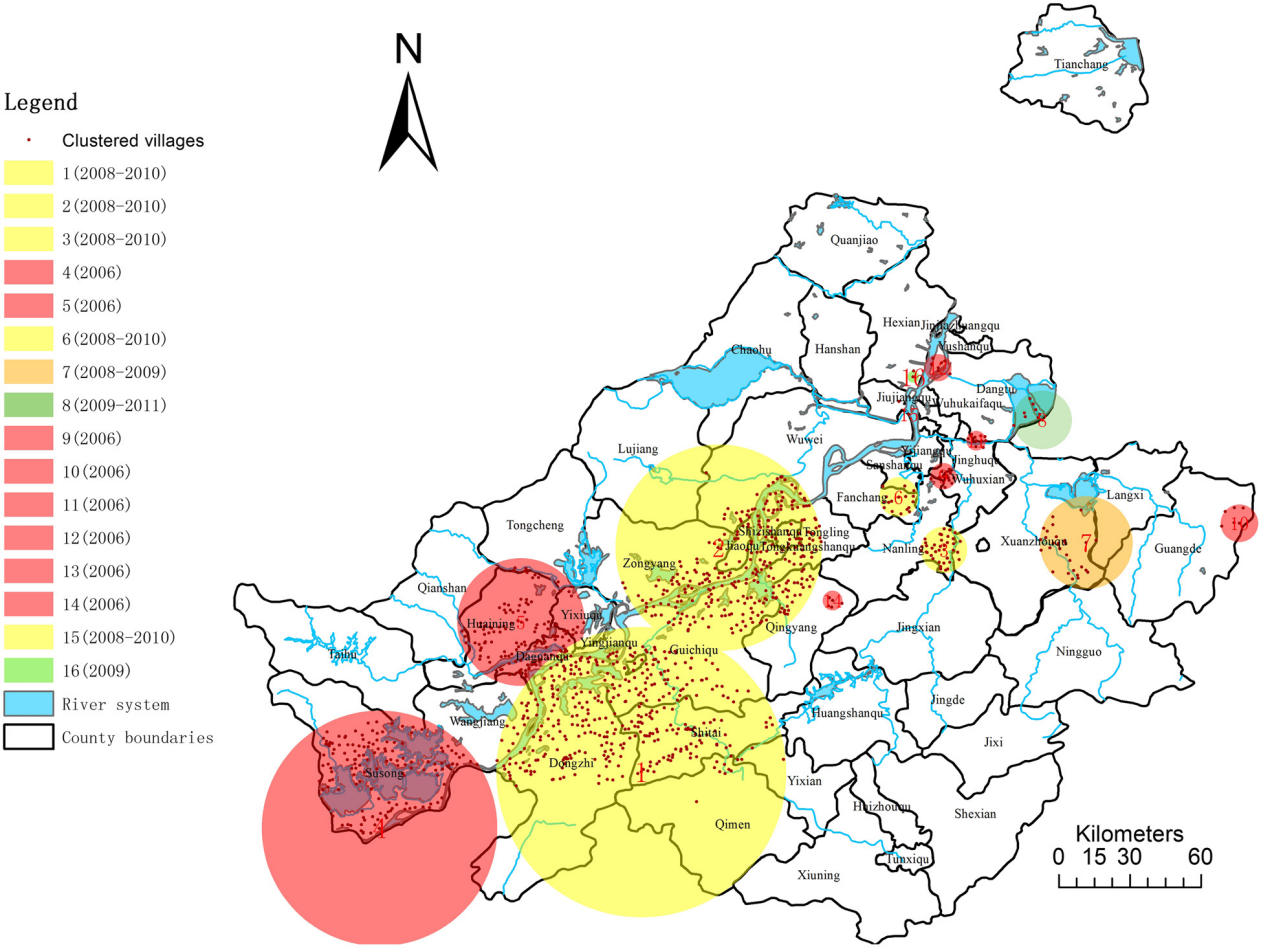
**16 space-time clusters of schistosomiasisinfection rate in human, using space-time scan statistics: Anhui province, 2006–2012.**

As shown in Figure 2 and Table 2, 16 clusters were observed in Anhui province from 2006 to 2012 with the biggest LLR being 10460 and the least 77 (p < 0.001), this indicates a statistically significant clustering pattern both in space and time in these areas. Regarding clustering pattern in time, there are eight foci observed in 2006 (purple circle), five in 2008–2010 (yellow circle), one in 2008–2009 (orange circle), one in 2009 (olive circle) and one in 2009–2011 (green circle). Results suggested that clustering of human infection with schistosomiasis are mainly found in 2006 while the trend tend to weaken over time.

**Table 2:** **Characteristics of detected clusters of infection rate of schistosomiasis in humans: Anhui province, 2006-2012**

**Cluster**	**Year**	**Cluster center**		**Radius(km)**	**No. of villages clustered**	**LLR**	**RR**	**(P-value)**
		**Latitude**	**Longitude**					
1	2008-2010	30.04946	117.30235	52.86	389	10460	3.47	<0.0001
2	2008-2010	30.89955	117.59288	37.74	308	4986	2.53	<0.0001
3	2008-2010	30.89118	118.45112	8.82	39	4047	4.65	<0.0001
4	2006	29.83447	116.30883	44.97	141	1972	2.8	<0.0001
5	2006	30.61691	116.84775	23.04	145	1526	2.99	<0.0001
6	2008-2010	31.09104	118.27803	7.37	17	1133	4.04	<0.0001
7	2008-2009	30.91815	118.98805	17.2	30	726	2.53	<0.0001
8	2009-2011	31.38328	118.82228	10.44	9	628	3.64	<0.0001
9	2006	31.17047	118.44757	4.8	13	516	5.22	<0.0001
10	2006	30.99129	119.57011	7.17	6	449	5.74	<0.0001
11	2006	31.30417	118.57212	3.84	10	307	4.78	<0.0001
12	2006	31.58058	118.42792	5.01	7	177	4.02	<0.0001
13	2006	31.45306	118.27908	0	1	93	6.55	<0.0001
14	2006	30.69838	118.02755	3.56	5	85	4.46	<0.0001
15	2008-2010	31.40428	118.31721	0	1	82	3.56	<0.0001
16	2009	31.54630	118.33275	2.46	2	77	5.15	<0.0001

Considering the geographical distribution, all 16 clustering foci were located along the Yangtze River and its connecting branches. Among these are three major clusters distributed along the upstream. Cluster 1 has the widest range including a total of 389 endemic villages in Dongzhi county, Guichiqu county and Shitai county, covering branches of Qiupu river and Shenjing Lake aside from the Yangtze River (LLR = 10460, RR = 3.47). Meanwhile, Cluster 4, which has an LLR of 1972 and RR of 2.8, contains 141 endemic villages in Susong county and only Yangtze River and its tributaries were covered in this area. The last one, Cluster 5, covers 145 endemic villages in Huaining county, Daguan county and Yixiu county (LLR = 1526, RR = 2.99) and two river branches in relation with the Yangtze River and Shimen Lake.

Besides, two clusters were observed in midstream of the Yangtze River. The bigger one, which has an LLR of 4986 and RR of 2.53 covers 308 endemic villages across four counties (Zongyang, Tongling, rural Tongling and Qingyang), while the smaller one only covers five endemic villages in Qingyang county (LLR = 85, RR = 4.46). In spite of that, seven clusters (clusters 6, 3, 9, 7, 11, 8 and 12) observed in the downstream of the Yangtze River are much smaller than their counterparts in the upstream as shown in Table 2.

Though less clusters were detected in upstream, they include 88% of the total endemic villages (988/1235), indicating a larger range of region and more severe infection in local residents, while the leftmost are relatively smaller with less villages included. Meanwhile, most clusters were detected in 2006 and from 2008 to 2010.

### Spatial temporal cluster analysis of prevalence of schistosomiasis in cattle

**Figure 3:**
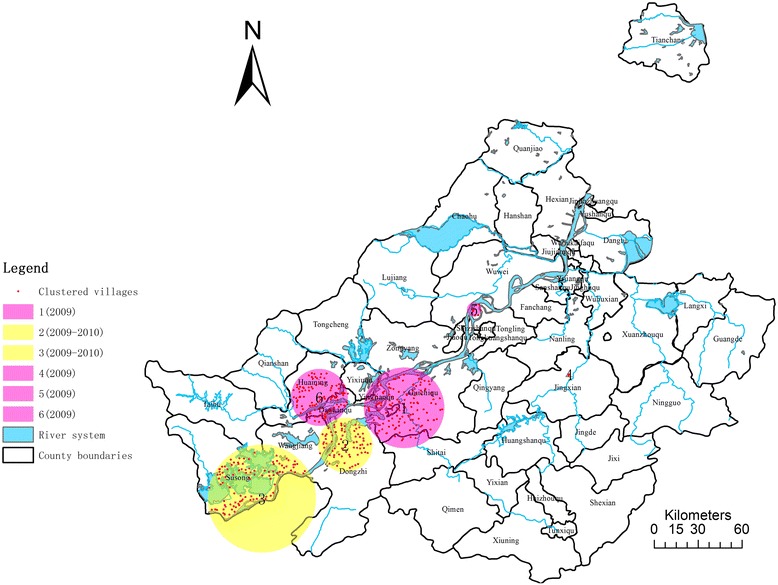
**Clusters of infection rate of schistosomiasis in cattle: Anhui province, 2009–2012.**

Results on infection rate in cattle found six statistically significant clusters from 2009 to 2012 in Anhui province (Figure 3 and Table 3), with the biggest LLR being 340 and smallest, 11 (p < 0.05). Temporally, four out of six appeared in 2009 (purple) while the other two showed in 2009–2010 (yellow). For geographic distribution, cluster 1 (LLR = 340,RR = 10.41), cluster 2 (LLR = 42, RR = 2.23), cluster 3 (LLR = 38,RR = 2.09) and cluster 6 (LLR = 11, RR = 1.97) are all located along the upstream of Anhui section of the Yangtze River and covered the most endemic villages, which is 132, 69, 102 and 110, respectively. While the two other clusters, cluster 4 (LLR = 19, RR = 17.73) and cluster 5 (LLR = 17, RR = 4.17), located along the midstream of Anhui section of the Yangtze River, only cover 2 and 9 villages.

**Table 3:** **Characteristics of detected clusters of prevalence of schistosomiasis in cattle: Anhui province, 2009-2012**

**Cluster**	**Year**	**Cluster center**		**Radius(km)**	**No. of villages clustered**	**LLR**	**RR**	**(P-value)**
		**Latitude**	**Longitude**					
1	2009	30.47433	117.34901	23.54	132	340	10.41	<0.0001
2	2009-2010	30.24420	116.98820	15.89	69	42	2.23	<0.0001
3	2009-2010	29.91628	116.47537	32.13	102	38	2.09	<0.0001
4	2009	30.67719	118.36205	0.85	2	19	17.73	<0.0001
5	2009	31.07630	117.77940	4.53	9	17	4.17	0.0007
6	2009	30.53713	116.82913	17.08	110	11	1.97	0.06

### Spatial temporal cluster analysis of occurrence rate of infected environments

**Figure 4:**
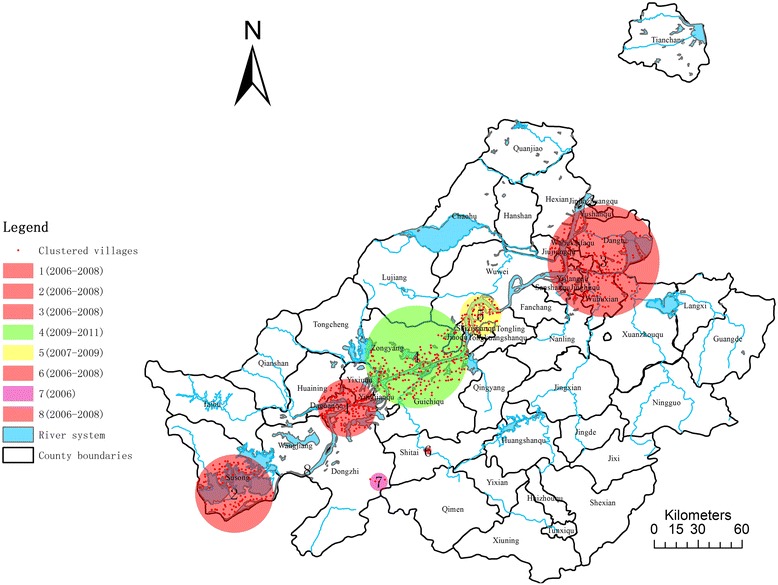
**Eight clusters of occurrence rate of infected environment using space-time scan statistics: Anhui province, 2006–2012.**

As shown in Figure 4 and Table 4, eight significant clusters of schistosomiasis infected environment were detected between 2006 and 2012 with the largest LLR being 348 and smallest, 12 (p < 0.05). However, five out of eight appeared in 2006–2008 (red), one in 2006 (purple), one in 2007–2009 (yellow) and one in 2007–2011 (green). Geographically, cluster 1, 2, 3 and 6, located along the up- and mid-stream of Anhui section of the Yangtze River, covered the most endemic villages with relatively bigger radius compared to their midstream counterparts.

**Table 4:** **Characteristics of detected clusters of occurrence rate of infected environment: Anhui province, 2006-2012**

**Cluster**	**Year**	**Cluster center**		**Radius(km)**	**No. villages clustered**	**LLR**	**RR**	**(P-value)**
		**Latitude**	**Longitude**					
1	2006-2008	30.47203	117.00040	16.96	75	348	13.1	<0.001
2	2006-2008	31.37622	118.57353	32.69	141	146	6.81	<0.001
3	2006-2008	29.94667	116.30667	21.07	29	129	12.3	<0.001
4	2009-2011	30.78597	117.42320	30.21	70	124	7.15	<0.001
5	2007-2009	31.04350	117.81800	12.42	59	24	4.47	<0.001
6	2006-2008	30.20934	117.49571	2.37	5	23	5.03	<0.001
7	2006	30.01853	117.19403	7.16	6	17	6.08	<0.001
8	2006-2008	30.09210	116.75587	1.59	4	12	3.62	0.045

### Spatial temporal cluster analysis of incidence rate of acute schistosomiasis

**Figure 5:**
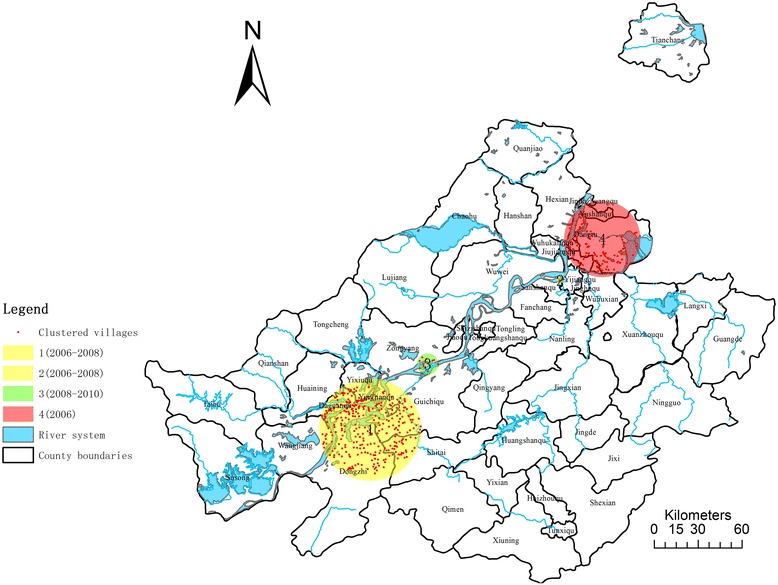
**Four clusters of incidence rate of acute schistosomiasis, using space-time statistics Anhui province, 2006–2012.**

Table 5 and Figure 5 show the clustering results of incidence rate of acute schistosomiasis. Only four clusters were found statistically significant (p < 0.05) with the LLR ranging from 12 to 30, which is less than the other endemic indicators.

**Table 5:** **Characteristicsof detected clusters of incidence rate of acute schistosomiasis: Anhui province, 2006-2012**

**Cluster**	**Year**	**Cluster center**		**Radius(km)**	**No. villages clustered**	**LLR**	**RR**	**(P-value)**
		**Latitude**	**Longitude**					
1	2006-2008	30.33722	117.14012	30.13	328	30	6.97	<0.0001
2	2006-2008	31.25450	118.30622	2.06	2	18	107.8	0.0002
3	2008-2010	30.74405	117.49400	6.07	12	16	26.59	0.0017
4	2006	31.50872	118.56687	22.06	104	12	11.8	0.038

Temporally, three out of four appeared before 2008 (red for 2006, yellow for 2006–2008) and only one was observed in 2008–2010 (green). Meanwhile, among the four clusters, upstream cluster 1 of Anhui section of the Yangtze River (LLR = 30, RR = 6.97, Radius = 30.13 km) and downstream cluster 4 of Anhui section of the Yangtze River (LLR = 12, RR = 11.8, Radius = 22.06 km) covers the most areas, with 328 and 104 endemic villages included, respectively. In spite of that, cluster 2 (LLR = 18, RR = 107.8) and cluster 3 (LLR = 16, RR = 26.59) only covered 2 and 12 villages, respectively.

### Spatial regression analysis of the independence of the human infection rate

The results of the spatial regression models are shown in Table 6. As described in Table 6, the human infection rate was significantly correlated with the other three indicators, and the spatial error model best fits both covariates. Y,x1,*x*2,x3 was used to reflect the human infection rate, cattle infection rate, occurrence rate of infected environments and incidence rate of acute schistosomiasis, respectively. By the spatial error model statistics, it showed a regression relationship by the following formula: Y = 0.035 * ×1 + 0.014 * ×2 + 22.34 * ×3.

**Table 6:** **Results of the spatial regression model: p-values are shown in brackets**

**Estimation**	**Ols**	**Spatial Lag**	**Spatial error**
Constant	0.2456 (0.0001)	−0.0032 (0.7749)	0.2086 (0.0012)
R^2^	0.2076 (0.0001)	0.3768^*^	0.3815^*^
Log likelihood	−1862.73	−1557.02	−1555.6
Akaike Inf. criterion	3733.46	3124.04	3119.13
Schwarz criterion	3757.1	3302.54	3142.78
Likelihood ratio test	^*^	670.77 (0.0001)	614.30 (0.0001)
LM lag	^*^	3038.82 (0.0001)	^*^
Robust LM lag	^*^	201.44 (0.0001)	^*^
LM error	^*^	^*^	3925.63 (0.0001)
Robust LM error	^*^	^*^	1088.25 (0.0001)
Breusch-Pagan test	209.13 (0.0001)	139.68 (0.0001)	120.19 (0.0001)

^*^Not available.

## Discussion

Anhui province was known as a schistosomiasis endemic hotspot in the past, this could be attributed to the varying complexity in the geographic landscape of the entire region and the interplay between human activities, cattle and vector snails that are important components in the epidemiology of the disease [7,23]. This complexity has enhanced the transmission of schistosomiasis in the region and also makes control strategies targeted at eliminating this debilitating disease in the region difficult to be effectively implemented [29,30]. However, should information on spatial and temporal clustering pattern of schistosomiasis infection in Anhui province be provided, future control strategies would be effectively implemented owing to the adequate background knowledge available [31]. Based on the annual surveillance of human infection, livestock infection and presence of infected vector snails in the different environments in Anhui province from 2006 to 2012, this study visualized the clustering of schistosomiasis endemic status in Anhui province by utilization of spatial-temporal analysis at village level.

The results showed a clustering pattern in infection rate of humans and cattle, occurrence rate of infected environment and the incidence rate of acute cases both across time and space, while the number of clusters being detected was 16, 6, 8 and 4, respectively. Most clusters detected are distributed along the Yangtze River and its connecting branches. Therefore, about 80% severely endemic counties are distributed along the Yangtze River and its connecting branches in Anhui province currently where schistosomiasis control efforts are difficult to be implemented in recent years.

Regarding the clustering of human and cattle infection, 88% and 97% clustered villages were distributed along the upper- and middle-stream of Anhui section of the Yangtze River, respectively. This distribution pattern reveals that the human and cattle infection with schistosomiasis are much more severe in the upper- and middle-stream of Anhui section of the Yangtze River. This finding corresponds with previous studies performed in Anhui and Jiangsu province [32,33]. Areas along the upper- and middle-stream of Anhui section of the Yangtze River and its connecting branches mainly belong to the lake region known to be endemic for schistosomiasis transmission [23,34]. This region is characterized by a mixture of lakes, rivers and marshlands with vector snails. The marshland usually dry up during winter and becomes flooded in the rainy season [35]. Cattle roaming on the marshland infected the snails breeding in the surrounding areas these result in the aggregation of endemic villages with infected environment [36]. People and other livestock in turn get infected during daily life activities by contact with water containing schistosome cercariae [37]. Consequently, cluster of human schistosomiasis cases is in close relationship with the cluster of cattle in endemic areas, and both cluster of acute schistosomiasis and infected environment spots are significantly related to each other, suggesting that acute schistosomiasis occurrence is predominant in areas within infected environment spots especially in clusters found in the upstream and downstream of Anhui section of the Yangtze River.

Three clusters of infected vector snails were found in the mountainous region during this study period. This region accounts for 10.8% of the total size of endemic areas in Anhui province, including Shitai, Xuanzhou, Jingxian county which are located along the areas far from the Yangtze water system. However, since, the main source of infection is wild animals in Shitai county it is more difficult to control schistosomiasis in this area [38], therefore, the prevalence of infected environment spots has remained at a relatively high level compared to neighboring regions for years despite the annual intensive mollusciciding exercises [39].

Among 16, 6, 8 and 4 clusters detected by using space-time scan statistics of infection rate of human and cattle, occurrence rate of infected environment and the incidence rate of acute cases 15, 6,7,4 clusters appeared before 2010, respectively. The declining trend of clusters detected over time and the profound decline in the tested endemic indicators of schistosomiasis suggests that the intensive integrated strategies on the control of schistosomiasis have received positive feedback [38,40]. The waning of endemic situation detected in recent years in Anhui province could be attributed to the increasing implementation of the national program on schistosomiasis control with strategy focusing on the control of infection sources since 2004 [5], the development of the Three Gorges Project in the upstream of the Yangtze River, which is responsible for the decreasing numbers of snails in the endemic areas downstream [41], and the transformation in the life style of local residents, which discourage contact with infested water, influenced the corresponding decline in the risk of being infected [24].

## Conclusion

In conclusion, this study identified the hotspots of endemic areas with accelerated risks both temporally and geographically at a fine scale from 2006 to 2012 in Anhui province based on the spatial-temporal analysis performed at village level, providing a scientific evidence for the future implementation of control strategies. However, only four indicators were taken into account in this study. The factors such as environmental variables, socio-cultural, and behavioral factors etc. were not included, as the previous study [42]. Hence, this study could not correlate schistosomiasis infection in human with other factors such as environmental variables and socio-cultural factors.
